# Direct Staining with Major Histocompatibility Complex Class II Dextramers Permits Detection of Antigen-Specific, Autoreactive CD4 T Cells *In Situ*

**DOI:** 10.1371/journal.pone.0087519

**Published:** 2014-01-27

**Authors:** Chandirasegaran Massilamany, Arunakumar Gangaplara, Ting Jia, Christian Elowsky, Guobin Kang, Jean-Jack Riethoven, Qingsheng Li, You Zhou, Jay Reddy

**Affiliations:** 1 School of Veterinary Medicine and Biomedical Sciences, University of Nebraska-Lincoln, Lincoln, Nebraska, United States of America; 2 Center for Biotechnology, University of Nebraska-Lincoln, Lincoln, Nebraska, United States of America; 3 Nebraska Center for Virology and School of Biological Sciences, University of Nebraska-Lincoln, Lincoln, Nebraska, United States of America; University of Utah School of Medicine, United States of America

## Abstract

We report here the utility of major histocompatibility complex (MHC) class II dextramers for *in situ* detection of self-reactive CD4 T cells in two target organs, the brain and heart. We optimized the conditions for *in situ* detection of antigen-specific CD4 T cells using brain sections obtained from SJL mice immunized with myelin proteolipid protein (PLP) 139–151; the sections were costained with IA^s^/PLP 139–151 (specific) or Theiler's murine encephalomyelitis virus (TMEV) 70–86 (control) dextramers and anti-CD4. Analysis of sections by laser scanning confocal microscope revealed detection of cells positive for PLP 139–151 but not for TMEV 70–86 dextramers to be colocalized with CD4-expressing T cells, indicating that the staining was specific to PLP 139–151 dextramers. Further, we devised a method to reliably enumerate the frequencies of antigen-specific T cells by counting the number of dextramer^+^ CD4^+^ T cells in the ‘Z’ serial images acquired sequentially. We next extended these observations to detect cardiac myosin-specific T cells in autoimmune myocarditis induced in A/J mice by immunizing with cardiac myosin heavy chain-α (Myhc) 334–352. Heart sections prepared from immunized mice were costained with Myhc 334–352 (specific) or bovine ribonuclease 43–56 (control) dextramers together with anti-CD4; the sections showed the infiltrations of Myhc-specific CD4 T cells. The data suggest that MHC class II dextramers are useful tools for enumerating the frequencies of antigen-specific CD4 T cells *in situ* by direct staining without having to amplify the fluorescent signals, an approach commonly employed with conventional MHC tetramers.

## Introduction

Traditionally, limiting dilution analysis, enzyme-linked immunosorbent spot assay, intracellular cytokine staining, and cytokine-secretion assay have been used to enumerate frequencies of antigen-specific T cells [1]–[7]. While all these are functional assays, they provide information largely at the population level. Additionally, low specificity and the laborious nature of these assays may limit their use for routine applications [1]–[5]. To overcome these limitations, and to be able to phenotype the antigen-specific T cells at a single cell level, major histocompatibility complex (MHC) tetramer technology has been developed [8]. The use of tetramers has revolutionized our understanding of the nature of immune responses with respect to the appearance, disappearance and/or persistence of antigen-specific T cells in experimental and clinical conditions [5], [9].

While MHC class I tetramers permit detection of antigen-specific CD8 T cells, MHC class II tetramers are used to analyze CD4 T cell responses. Detection of antigen-specific CD4 T cells using MHC class II tetramers, however, is particularly challenging compared to using MHC class I tetramers [5], [9]–[14]. Several factors contribute to this disparity: 1) low affinity of MHC class II/peptide complexes; 2) low affinity of T cell receptors (TCRs) for MHC/peptide complexes; 3) instability of soluble MHC class II monomers in *in vitro* expression systems; 4) improper registry and geometry of peptides for display by MHC molecules; 5) lack of participation of CD4 coreceptors in MHC-binding; and 6) activation-dependency [13], [15]–[18]. In our efforts to improve the sensitivity of MHC class II tetramers, we recently created next-generation tetramers, designated “dextramers.” The dextramer reagents proved helpful in enumerating the frequencies of autoreactive CD4 T cells in several murine autoimmune disease models as evaluated by flow cytometry [19].

Structurally, dextramers contain dextran backbones, which are polymers of glucose molecules attached through 1–6 and 1–3 linkages [20]. Each dextran molecule carries multiple moieties of streptavidin to which biotinylated peptide-tethered MHC molecules can be assembled [20]. As a result, MHC dextramers contain aggregates of MHC-peptide complexes, allowing them to engage multiple TCRs – more than that could be achieved with tetramers. Using three different autoimmune disease models − myelin proteolipid protein (PLP) 139-151-induced experimental autoimmune encephalomyelitis (EAE) in SJL mice; myelin oligodendrocyte glycoprotein (MOG) 35-55-induced EAE in C57Bl/6 mice; and cardiac myosin heavy chain-α (Myhc) 334-352-induced experimental autoimmune myocarditis (EAM) in A/J mice − we demonstrated that the MHC class II dextramers were at least five-fold more sensitive than the tetramers, and their specificity was also superior [19]. In this study, using PLP 139-151-induced EAE and Myhc 334-352-induced EAM models, we report that MHC class II dextramers can be successfully used to detect autoreactive CD4 T cells *in situ* with a high degree of specificity by direct staining without the need to amplify the signals with fluorophore antibodies, which is generally required with tetramers. We also describe a comprehensive method of evaluating tissues to accurately enumerate the frequencies of antigen-specific CD4 T cells *in situ*.

## Materials and Methods

### Ethics statement

Five-to-six-week-old female SJL/J (H-2^s^) and six-to-eight-week-old female A/J mice were obtained from the Jackson Laboratory (Bar Harbor, ME, USA). The mice were maintained in accordance with the animal protocol guidelines of the University of Nebraska-Lincoln, Lincoln, NE, USA. The study was conducted in accordance with National Institutes of Health guidelines for the use of experimental animals, and the protocols were specifically approved by The University of Nebraska-Lincoln Institutional Animal Care and Use Committee (permit number: A3459-01; protocol # 659 and 615).

### Peptide synthesis and immunization procedures

PLP 139–151 (HSLGKWLGHPDKF), and Myhc 334–352 (DSAFDVLSFTAEEK AGVYK) were synthesized on 9-fluorenylmethyloxycarbonyl chemistry (Neopeptide, Cambridge, MA, USA). All peptides were HPLC-purified (>90%), identity-confirmed by mass spectroscopy, and dissolved in sterile 1× PBS prior to use. Peptides were then emulsified in complete Freund's adjuvant (CFA) supplemented with *Mycobacterium tuberculosis* (Mtb) H37RA extract (Difco Laboratories, Detroit, MI, USA) to a final concentration of 5 mg/ml, and the emulsions were administered s.c. to induce EAE or EAM as we have described previously [19], [21], [22]. To generate primary T cell cultures, peptides emulsified in CFA containing Mtb (1 mg/ml) were administered s.c. (100 µg/mouse) as described previously [19], [22].

### Clinical scoring and collection of tissues for in situ staining

SJL mice immunized with PLP 139–151 were monitored for clinical signs of EAE and scored as described previously [22], [23]. The animals reaching scores of 3 and above were euthanized using a prefilled CO_2_ chamber, and the left ventricles of their hearts were perfused with 10 ml of cold 1× PBS. Cerebrums were gently excised by blunt dissection and placed in cold 1× PBS. Similarly, on day 21 postimmunization with Myhc 334–352, A/J mice were euthanized and perfused as above, and hearts were collected and placed in cold 1× PBS.

### Generation of MHC class II dextramers

IA^s^ dextramers (PLP 139–151 and Theiler's murine encephalomyelitis virus [TMEV] 70–86) and IA^k^ dextramers (Myhc 334–352 and bovine ribonuclease [RNase] 43–56) were generated as we have described previously [19]. Briefly, α and β constructs for each IA allele containing the sequences of the respective peptides were expressed in baculovirus using Sf9 insect cells (Invitrogen, Carlsbad, CA), and the soluble monomers of IA^s^ and IA^k^ were purified [22], [24], [25]. After being concentrated, the soluble MHC class II proteins were biotinylated using biotin protein ligase at an optimized concentration of 25 µg/10 nmol of substrate as recommended by the manufacturer (Avidity, Denver, CO). To prepare dextramers, the biotinylated proteins were mixed with activated dextran backbones (kindly provided by Immudex, Copenhagen, Denmark) at a molar ratio of 20∶1 in 1 x Tris HCl 0.05 M, pH 7.2, for 30 minutes at room temperature (RT). The reagents were aliquoted and stored at 4°C until use.

### Generation of antigen-sensitized primary T cell cultures and dextramer staining

Draining lymph nodes (mandibular, axillary and inguinal) were collected from immunized mice. After the single cell suspensions were prepared, the lymph node cells (LNC) were stimulated with the immunizing peptides (PLP 139–151, 20 µg/ml or Myhc 334–352, 50 µg/ml) at a density of 5×10^6^ cells/ml for two days, and interleukin (IL)-2 medium was then added [21], [22], [24]. Cells were stained with dextramers on the indicated days in IL-2 medium, pH 7.6, containing 2.5% fetal bovine serum at RT for 2 hours, followed by staining with anti-CD4 (eBioscience, San Diego, CA) [19]. After cells were washed and fixed with 0.5% PBS-buffered 4% paraformaldehyde, they were examined under the laser scanning confocal microscope (LSCM). Similarly, PLP 139–151-specific T cell hybridoma clone (B8) generated in our laboratory was also stained and examined as above.

### Tissue sectioning using vibratome

Cerebrums and hearts were embedded in molten 4% PBS-buffered low-melting agarose (40°C). To facilitate transverse sectioning, the cerebrums were embedded with the ventral surface facing downward, as opposed to the base facing downward for hearts. The agarose-embedded tissues were patted dry and mounted onto a vibratome platform, with the ventral surface of the cerebrum or the base of the heart attached with Loctite super glue. After tissues were immobilized, the platform was locked in the vibratome bath filled with cold 1× PBS. A clean razor blade was attached to the vibratome with the blade angle set at 15°, and 200 µm thick sections were made sequentially at a dead slow speed “0.22 mm/sec.” Sections were then transferred into a 24-well plate containing cold 1× PBS. For quantitative analysis of dextramer^+^ (dext^+^) cells in the brains, three paired sections were cut from the dorsal to the ventral surface with a difference of 1.5 mm between pairs. Likewise, eight paired sections were made continuously from the hearts.

### Direct in situ staining with MHC class II/IA^s^ and IA^k^ dextramers

We performed dextramer staining in 24-well plates. The cerebral sections were stained individually, but up to three heart sections were stained together in each well because they were small. Sections were stained in 300 µl of blocking solution (1 x PBS/2% normal goat serum) containing dextramer-allophycocyanin (APC; 2.5 µg/ml) and anti-CD4-phycoerythrin (PE; 5 µg/ml), and incubated in the dark at RT for 1.5 hours on a rocking shaker with gentle rotations. The cocktails of reagents used for cerebral sections were either IA^s^/PLP 139–151 dextramers/anti-CD4 or TMEV 70–86 dextramers/anti-CD4; for hearts they were either IA^k^/Myhc 334–352 dextramers/anti-CD4 or RNase 43–56 dextramers/anti-CD4. When staining was complete, the sections were washed three times with 1 ml cold 1x PBS and incubated at RT for 10 min on a rocking shaker for each wash. Sections were then fixed in 1 ml filtered 4% PBS-buffered paraformaldehyde (pH 7.4) at RT for 1.5 hours, followed by washing three times with cold 1x PBS as above, and mounted on clean glass slides using the mounting medium (Dako, Carpinteria, CA).

### Laser scanning confocal microscopy

For scanning and acquisition of images, we used the Nikon A1-Eclipse 90i confocal microscope system (Nikon Instruments Inc-Americas, Melville, NY). We used two channels to acquire the images sequentially under high magnification (100x) and their excitation/emission wavelengths were: 561.5 nm/553–618 nm laser for pseudocolored “green” channel (CD4-PE); and 640.7 nm/663–738 nm for pseudocolored “red” channel (dextramer-APC). The cells showing co-localization of signals generated from both the red (dextramers) and green (CD4) channels were identified as dext^+^ CD4^+^ T cells. To quantitatively analyze dext^+^ CD4^+^ T cells, we used the Olympus FV500-BX60 confocal microscope system (Olympus America Corporation, Central Valley, PA), in which sequential images were acquired using two channels: the Cy3 laser (543 nm, pseudocolored “green” for CD4-PE) and the Cy5 laser (633 nm, pseudocolored “red” for dextramer-APC). The sequential acquisition involved capturing images separately, based on the green signal (CD4-PE) followed by the red signal (dextramers-APC), then merging them. The quantitative analysis was performed in each mouse using three sets of paired sections from the cerebrum or eight sets of paired sections from the heart; one section in each set was stained with specific or control dextramers together with anti-CD4, and the sections were examined by LSCM. Initially, the sections were scanned under low magnification (4x) using the Cy3 laser alone (for CD4) to locate the spectrum of inflammatory foci across the surface. We then used both the Cy3 (for CD4) and Cy5 (for dextramers) lasers under high magnification (100x) to acquire ‘Z’ serial images sequentially at 2 µm intervals. In each cerebral section, 10 to 15 inflammatory foci were examined, and in each focus, a set consisting of 15 to 25 serial images was acquired. In contrast, a total of 20 inflammatory foci representing all eight sections in the hearts were subjected to ‘Z’ imaging, and 10 to 15 serial images were sequentially acquired from each focus. In all the images, ‘ImageJ’ software was used to count dext^+^ CD4^+^ T cells in relation to the total number of CD4^+^ T cells. Finally, the number of cells in all the ‘Z’ serial images representing three cerebral sections or eight heart sections were added to obtain the total number.

### Statistical analysis

We used MATLAB® (version 8.1.0.604 [R2013a], The MathWorks™) for statistical calculations involving cerebral sections. To compare the differences in dext^+^ CD4^+^ cells in the cerebral sections stained with PLP 139–151 dextramers or TMEV 70–86 dextramers, we used the non-parametric Wilcoxon signed-rank test. Student's *t*-test was used to determine differences in dext^+^ CD4^+^ cells in the heart sections stained with Myhc 334–352 dextramers or RNase 43–56 dextramers. p≤0.05 values were considered significant.

## Results and Discussion

Unlike MHC class I tetramers, the use of MHC class II tetramers for routine applications continues to be challenging, especially to enumerate the precursor frequencies of low-affinity autoreactive CD4 T cells, due in part to their activation dependency [9], [25]–[27]. Recently we circumvented this problem by creating the next generation of tetramers, called “dextramers,” permitting us to detect antigen-specific CD4 T cells for a number of autoantigens, such as PLP 139–151, MOG 35–55 and Myhc 334–352 [19]. Additionally, the creation of dextramer reagents requires a significantly low amount of MHC class II monomers. Once prepared, the reagents are stable for an extended period of time at 4°C [19]. Based on these successes, we made efforts to optimize conditions to detect antigen-specific self-reactive CD4 T cells *in situ* by direct staining with the dextramers.

**Figure 1:**
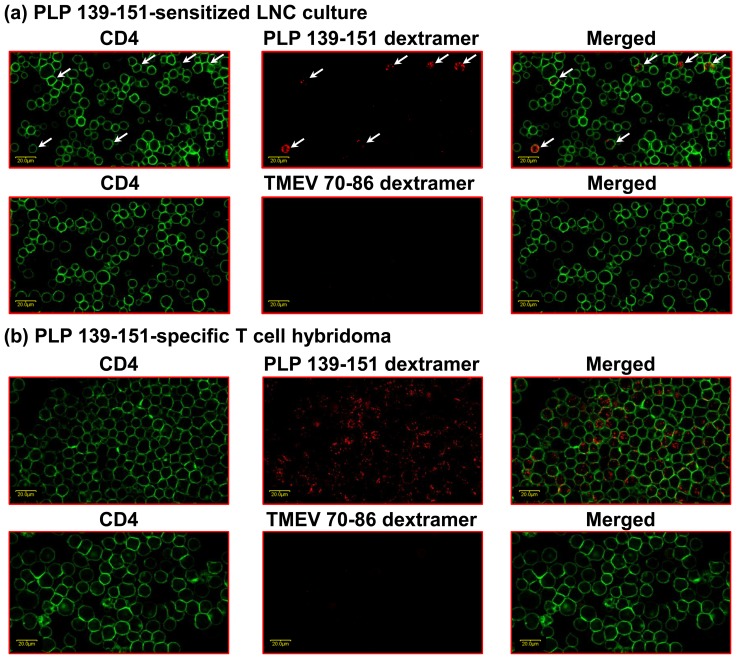
Evaluation of the specificity of PLP 139–151 dextramers by confocal microscopy *in vitro*. For dextramer staining, two cell sources were used: (a) PLP 139-151-sensitized LNCs obtained from immunized mice; and (b) PLP 139-151-specific T cell hybridoma clone (B8). Viable cells harvested from LNC cultures on day 6 poststimulation, and also hybridoma cells were stained with PLP 139–151 dextramers/anti-CD4 or TMEV 70–86 (control) dextramers/anti-CD4. After washing, cells were fixed with 0.5% paraformaldehyde and examined by LSCM. Top panels (panels, a and b): cells stained with PLP 139–151 dextramers and anti-CD4. Bottom panels (panels, a and b): cells stained with TMEV 70–86 dextramers (control) and anti-CD4. Left panels: CD4, green; Middle panels: dextramers, red; Right panels: merged. Dext^+^ CD4^+^ T cells are shown with arrows for LNCs, whereas in PLP 139-151-specific hybridoma cells, the dext^+^ CD4^+^ cells are shown as such throughout the field. Original magnification 600×; bar  = 20 µm.

We standardized the reaction conditions for visualization of self-reactive CD4^+^ T cells by LSCM using PLP 139–151 dextramers. Generally, two major factors can negatively influence the detection of antigen-specific T cells *in situ* by LSCM. First, insufficient signals emitted by fluorophores can lead to the need to amplify the signals using fluorophore antibodies. Second, such an indirect staining can undermine the specificity, as the background staining for control reagents also can increase proportionately [28], [29]. We overcame these limitations by direct staining with the dextramers. We used two cell sources for dextramer staining: (i) PLP 139-151-sensitized LNCs obtained from immunized animals; and (ii) PLP 139-151-specific T cell hybridoma. Briefly, cells were stained with PLP 139–151 or TMEV 70–86 (control) dextramers and anti-CD4 as described previously [19]; and after fixation, examined the dextramer-stained cells by LSCM. Figure 1 shows cells stained with anti-CD4 alone (left panels, green), with dextramers alone (middle panels, red) and with the merged CD4 and dextramers (right panels, yellow/red). As expected, the LNCs or hybridoma cells that appeared yellow/red in the merged panels also were stained with anti-CD4 and PLP 139–151 dextramers (Figure 1a and 1b, top panels). The punctate appearance of dext^+^ cells may represent aggregates of multimeric MHC/peptide complexes that interact with multiple TCRs [30]. Since such staining was lacking with TMEV 70–86 dextramers (control: Figure 1a and 1b, bottom panels), the data suggested that the staining obtained with PLP 139–151 dextramers was specific. Thus, the dextramers could be used to visualize antigen-specific T cells under LSCM by direct staining without having to amplify the signals with secondary fluorophore antibodies.

**Figure 2:**
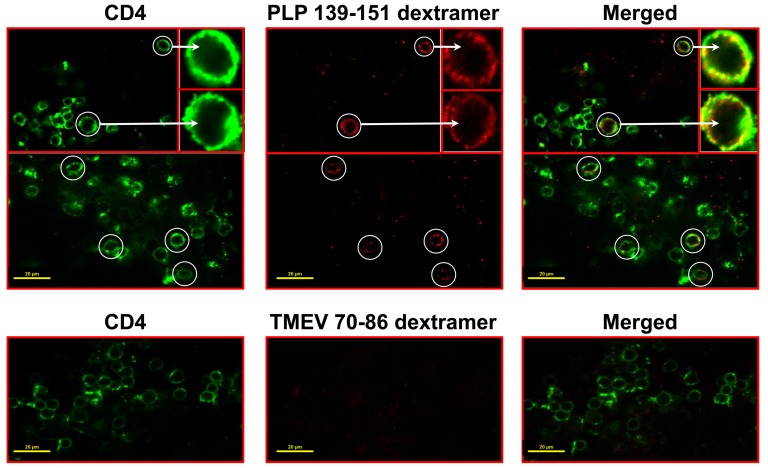
Detection of PLP-specific T cells by *in situ* staining with PLP 139–151 dextramers. EAE was induced in SJL mice by immunizing the animals with PLP 139–151 in CFA. At termination, cerebrums collected from EAE mice were embedded in 4% agarose, and the sections were made using vibratome. After staining with cocktails containing either PLP 139–151 dextramers/anti-CD4 or TMEV 70–86 dextramers (control)/anti-CD4 and fixing with 4% PBS-buffered paraformaldehyde, sections were washed and mounted for examination by LSCM. Top panels: sections stained with PLP 139–151 dextramers and anti-CD4. Bottom panels: sections stained with TMEV 70–86 dextramers and anti-CD4. Left panel: CD4, green; Middle panel: dextramers, red; Right panel: merged (circles, dext^+^ CD4^+^ T cells; insets represent enlarged views of dext^+^ CD4^+^ T cells). Original magnification 1000×; bar  = 20 µm.

We next sought to determine whether dextramers could be used to detect antigen-specific T cells in tissues. In brief, freshly cut cerebral sections were derived from EAE mice and stained with mixtures containing PLP 139–151 (specific) or TMEV 70–86 (control) dextramers and anti-CD4. The standardization experiments included two parameters, temperature (4°C, 37°C and RT) and duration of staining (30 min and 1.5 hours). We noted that the dextramer staining was found to be better at RT than 4° or 37°C with respect to the staining intensity and/or specificity (data not shown). Since a concentration of 2.5 µg/ml of PLP 139–151 dextramers incubated for 1.5 hours at RT yielded reproducible detection of PLP-specific CD4 T cells with no or negligible background staining for control dextramers, we chose these conditions for further experimentation. As shown in figure 2 (top panels), the LSCM analysis of brain sections costained with PLP 139–151 dextramers (red) and anti-CD4 (green) revealed the presence of yellow-colored double positive cells, a feature that was lacking in sections stained with control (TMEV 70–86) dextramers (bottom panels). Consistent with the appearance of dext^+^ CD4^+^ cells noted in the *in vitro*-stimulated cultures (Fig 1), the double-positive cells (dext^+^ CD4^+^) in the tissue sections also showed punctate dots around the surface (Fig 2, insets in the top panels). Since each dextran-APC molecule in the dextramers contained 9 to 10 MHC/peptide complexes as opposed to a maximum of four MHC/peptide complexes in conventional tetramers [19], staining with dextramers might have facilitated cross-linking of MHC/peptide complexes with multiple TCRs, leading to their enhanced avidity and better stability of TCR-MHC/peptide complexes. Such an engagement also may ensure that dextramers cannot be easily dislodged during a series of washing steps and, as a result, can be retained at the cell surface for an extended period of time. Additionally, on a reaction basis, dextramers offer the advantage of requiring a small amount of MHC/peptide complexes. In our protocol, each reaction requires only 0.75 µg of MHC/peptide monomers. Above all, staining with dextramers is a one-step reaction since dextramers are coincubated with anti-CD4, and the whole procedure, from tissue sectioning to staining, can be finished in less than a day. In contrast, the published protocols for *in situ* staining with conventional tetramers commonly involve amplification procedures in which the fluorescent signals are amplified using secondary antibodies for fluorophores. As a result, staining procedures may take up to three days to complete [28], [29], [33].

**Figure 3:**
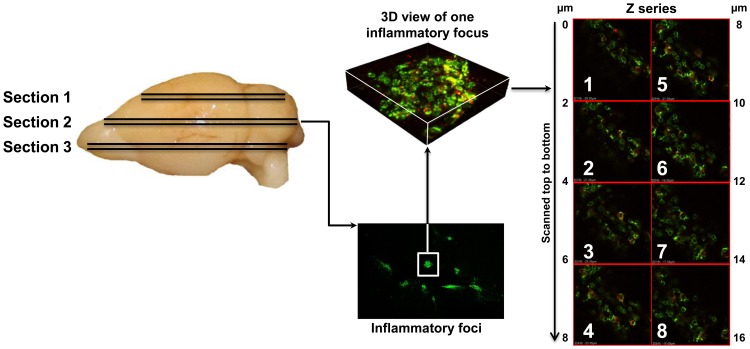
Quantitative analysis of PLP-specific CD4 T cells detected by *in situ* staining with PLP 139–151 dextramers in EAEmice. Three paired cerebral sections, each 200 µm thick, were made from brains obtained from EAEmice using vibratome by cutting the sections from the dorsal to the ventral surface with 1.5 mm intervals between pairs. One section from each pair was stained with either PLP 139–151 or TMEV 70–86 (control) dextramers together with anti-CD4. After fixing and mounting, the sections were examined by LSCM (60X), and each inflammatory focus was subjected to a set of ‘Z’ serial images (15 to 25) taken sequentially. In total, 10 to 15 sets of such ‘Z’ serial images representing as many inflammatory foci were acquired from each section. In all the images, cells positive for both PLP 139–151 dextramers and CD4 or CD4 alone were counted using ‘ImageJ’ software. Finally, the cells counted in all the ‘Z’ serial images obtained from all the inflammatory foci were added to obtain the total number per section.

To quantitatively analyze the frequencies of antigen-specific T cells, we then devised a method to count dext^+^ cells in the multiple images acquired serially. This analysis involved four steps: 1) As depicted in figure 3, three paired sections were made from the cerebrum, and one section from each pair was stained with PLP 139–151 dextramers/anti-CD4, and the other with control (TMEV 70–86) dextramers/anti-CD4. 2) In each section, 10 to 15 inflammatory foci were located based on staining with CD4 antibody (Fig 3, middle lower panel). 3) Each focus as visualized in the 3D view (Fig 3, middle upper panel) was subjected to a set of 15 to 25 ‘Z’ serial images, which were captured sequentially from top to bottom at 2 µm intervals (Fig 3, right panel). Essentially, 10 to 15 such sets of ‘Z’ serial images representing as many inflammatory foci were analyzed in each section. 4) In each image, the cells positive for CD4 antibody alone (green) and those showing colocalization of signals derived from both dextramers (red) and CD4 (green) and appearing as punctate yellow cells were counted by marking them separately using ImageJ software. Finally, a grand total was obtained for each section by adding the number of cells counted in all the ‘Z’ serial images, as shown in table 1. Since marking cells eliminated the possibility of recounting the same cells in multiple overlapping images, dext^+^ cells could be reliably enumerated in relation to the total number of CD4 T cells counted in each section.

**Table 1:** Quantitative analysis of PLP 139–151-dext^+^ CD4^+^ T cells in the brains of EAE mice by confocal microscopy.

Animal	PLP 139–151*	TMEV 70–86*
Mouse # 1		
Section 1	15/1245 (1.2%)	0/1007 (0%)
Section 2	10/770 (1.4%)	1/1033 (0.097%)
Section 3	3/322 (0.93%)	0/804 (0%)
Total	28/2337 (1.2%)	1/2844 (0.035%)
Mouse # 2		
Section 1	13/659 (1.97%)	0/609 (0%)
Section 2	9/689 (1.31%)	0/524 (0%)
Section 3	53/956 (5.5%)	0/937 (0%)
Total	75/2304 (3.3%)	0/2070 (0%)
Mouse # 3		
Section 1	11/1370 (0.8%)	0/301 (0%)
Section 2	3/325 (0.92%)	0/213 (0%)
Total	14/1695 (0.8%)	0/514 (0%)

* Number of dext^+^ CD4^+^ T cells/total number of CD4^+^ T cells

The data obtained from three individual mice indicate that the detection of PLP 139–151 dext^+^ cells varied between animals (0.8% to 3.3%) and between sections in each animal (0.8% to 5.5%; Table 1). Such variations were expected, as the inflammatory foci were scattered inhomogeneously across the section. Nonetheless, by counting the number of cells stained with control dextramers in parallel sections, we found only one cell out of a total of 5428 CD4^+^ cells to be positive for TMEV 70–86 dextramers (0.018%), as opposed to 117 CD4^+^ T cells found to be positive for PLP 139–151 dextramers (117/6336 = 1.85%; p = 0.0078). Based on flow cytometric analysis, we had previously reported detection of similar numbers of PLP 139–151 dext^+^ cells (2.3%) in mononuclear cells (MNCs) obtained from the brains of EAEmice, but the background staining for TMEV 70–86 dextramers also was high (0.7%; [19]). Thus, we have demonstrated that direct staining with dextramers allows antigen-specific, self-reactive CD4 T cells to be detected *in situ* in the brain. Notably, the use of dextramers has helped us detect antigen-specific T cells deep in the tissue sections, even up to 50 µm. However, our attempts to further enhance the detection sensitivity of dextramers by indirect staining using fluorophore antibodies to amplify the fluorescent signals yielded a very high background for control dextramers leading us to discontinue using this protocol in our routine experimentation (data not shown).

**Figure 4:**
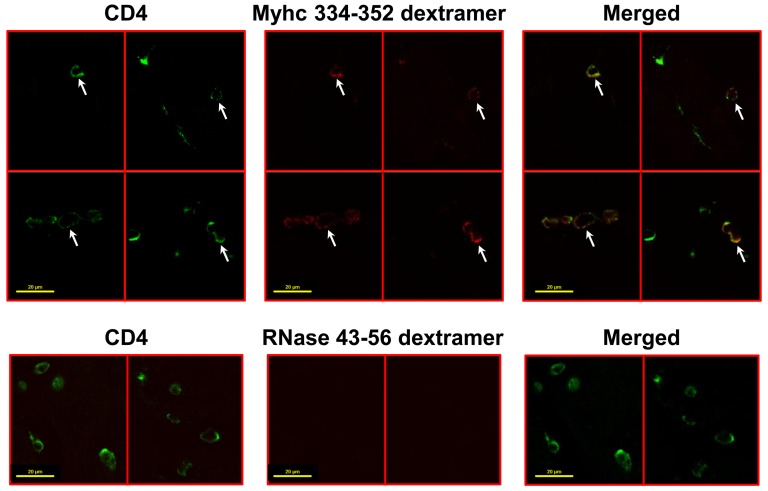
Detection of cardiac myosin-specific CD4 T cells by *in situ* staining. EAM was induced in A/J mice by immunizing the animals with Myhc 334–352 in CFA; at termination on day 21, hearts were collected after perfusion, and the tissue blocks were prepared using 4% PBS-buffered agarose. Eight paired sections of 200 µm thickness each were made using vibratome. One section from each pair (a total of eight sections) were stained with mixtures containing either Myhc 334–352 dextramers/anti-CD4 or RNase 43–56 (control) dextramers/anti-CD4. After fixing with 4% PBS-buffered paraformaldehyde, the sections were washed and mounted for examination by LSCM. A collage of images showing the cells positive for both dextramers and CD4 or CD4 alone is shown. Top panels: sections stained with Myhc 334–352 dextramers and anti-CD4. Bottom panels: sections stained with RNase 43–56 dextramers and anti-CD4. Left panel: CD4, green; middle panel: dextramers, red; right panel: merged. (arrows, dext^+^ CD4^+^ T cells). Original magnification 1000×; bar  = 20 µm.

We further extended the utility of dextramers for *in situ* detection of antigen-reactive T cells by adopting the Myhc 334-352-induced EAM model in A/J mice, in which the disease is primarily mediated by T cells [31], [32]. To evaluate antigen specificity, we derived LNC cultures from mice immunized with Myhc 334–352. Using RNase 43–56 dextramers as controls, we confirmed that Myhc 334–352 dextramer-bound CD4^+^ T cells could be visualized by LSCM with specificity (data not shown). We next analyzed the ability of Myhc 334–352 dextramers to detect Myhc-reactive T cells *in situ*, which were expected to infiltrate into the hearts as we had demonstrated previously by flow cytometry [19]. To this end, we obtained eight paired sections, each 200 µm thick, from A/J mice immunized with Myhc 334–352. One section from each pair (a total of eight sections) were costained with Myhc 334–352 dextramers/anti-CD4; the other set of eight sections were costained with RNase 43–56 dextramers (control)/anti-CD4. A total of 20 sets of ‘Z’ serial images representing an equal number of inflammatory foci from all eight sections were sequentially acquired as described above. In essence, each set of ‘Z’ serial images contained 10 to 15 individual images, each representing one individual focus of inflammation. LCSM analysis of these images revealed the presence of Myhc 334–352 dext^+^ cells (red) colocalized with the expression of CD4 (green) appearing as yellow cells containing punctae as expected (Fig 4, top panels). By pooling the dext^+^ CD4^+^ cells in all eight sections from each mouse, we noted frequencies of Myhc 334–352 dext^+^ cells in the range of 0.92% to 2.0%, as opposed to 0% to 0.09% for control dextramers (1.52±0.32% vs. 0.05±0.03%, p = 0.01; Table 2). Although the number of Myhc 334–352 dext^+^ cells detected by the *in situ* technique was marginally lower than the number that could be detected by flow cytometric analysis of MNCs obtained from heart-infiltrates (1.52% vs. 2.34%, [19]), the background staining for control dextramers was negligible with *in situ* detection (Fig 4; 0.05% vs. 0.34%, [19]). We thus proved the superiority of using Myhc 334–352 dextramers for reliable quantification of antigen-specific CD4 T cells *in situ*. However, in contrast to the signal intensity obtained with the PLP 139–151 dextramers in cerebral sections (Fig 2), the signal intensity obtained with Myhc 334–352 dextramers was low in heart sections, possibly due to the autofluorescence emitted by the heart tissue. This variation may be due to the structural/anatomical characteristics of tissues in different organs. For example, CNS tissues contain a high amount of lipids, whereas heart tissue is predominantly formed by striated muscle fibers, which may limit the diffusion of dextramers easily into the infiltrates. Consistent with this notion, we could detect Myhc 334–352 dext^+^ cells at a depth only up to 20 µm in most heart sections. In addition, we noted that Myhc 334–352 dext^+^ CD4^+^ T cells were scattered throughout the myocardium, indicating that the infiltrates were diffuse in myocarditic lesions.

**Table 2:** Quantitative analysis of Myhc 334–352 dext^+^ CD4^+^ T cells in the hearts of EAM mice by confocal microscopy.

Animal	Myhc 334–352*	RNase 43–56*
Mouse # 1	34/1699 (2.0%)	1/1313 (0.08%)
Mouse # 2	21/2282 (0.92%)	1/1157 (0.09%)
Mouse # 3	26/1571 (1.65%)	0/1128 (0%)

* Number of dext^+^ CD4^+^ T cells/total number of CD4^+^ T cells.

In conclusion, our data indicate that MHC class II dextramers can be successfully used to detect antigen-specific CD4 T cells *in situ* by direct staining. To our knowledge, we are the first to report the use of MHC class II dextramers to detect autoreactive CD4 T cells *in situ*. Furthermore, our method of analyzing antigen-specific CD4 T cells involves considerably less time – the whole procedure can be completed in less than a day, as opposed to three days with previously published protocols [28], [29], [33]. Additionally, our protocol requires low amount of starting MHC/peptide complexes (0.75 µg/reaction), and the dextramers have high degree of specificity and sensitivity. Although we have successfully demonstrated the utility of MHC class II dextramers for detection of antigen-specific CD4 T cells using fresh sections, the utility of these reagents in detecting cells in frozen tissues remains to be tested. Such an endeavor can be technically challenging because of difficulties experienced by other investigators in retaining the integrity of tissues during staining procedures [28], [29].
